# Internet-based cognitive rehabilitation for working cancer survivors: results of a multicenter randomized controlled trial

**DOI:** 10.1093/jncics/pkad110

**Published:** 2024-01-25

**Authors:** Kete M Klaver, Saskia F A Duijts, Chantal A V Geusgens, Jacobien M Kieffer, Joost Agelink van Rentergem, Mathijs P Hendriks, Janine Nuver, Hendrik A Marsman, Boelo J Poppema, Tanja Oostergo, Annemiek Doeksen, Maureen J B Aarts, Rudolf W H M Ponds, Allard J van der Beek, Sanne B Schagen

**Affiliations:** Department of Psychosocial Research and Epidemiology, Netherlands Cancer Institute, Amsterdam, the Netherlands; Department of Public and Occupational Health, Amsterdam University Medical Center location Vrije Universiteit, Amsterdam, the Netherlands; Amsterdam Public Health Research Institute, Societal Participation and Health, Amsterdam, the Netherlands; Department of Public and Occupational Health, Amsterdam University Medical Center location Vrije Universiteit, Amsterdam, the Netherlands; Amsterdam Public Health Research Institute, Societal Participation and Health, Amsterdam, the Netherlands; Department of Research and Development, Netherlands Comprehensive Cancer Organisation (IKNL), Utrecht, the Netherlands; Department of Medical Psychology, Amsterdam University Medical Center location Vrije Universiteit, Amsterdam, Amsterdam, the Netherlands; Department of Medical Psychology, Zuyderland Medical Center, Sittard, the Netherlands; Department of Psychosocial Research and Epidemiology, Netherlands Cancer Institute, Amsterdam, the Netherlands; Department of Psychosocial Research and Epidemiology, Netherlands Cancer Institute, Amsterdam, the Netherlands; Department of Medical Oncology, Northwest Clinics, Alkmaar, the Netherlands; Department of Medical Oncology, University Medical Center Groningen, Groningen, the Netherlands; Department of Surgery, OLVG, Amsterdam, the Netherlands; Department of Medical Oncology, Ommelander Hospital Group, Groningen, the Netherlands; Department of Medical Oncology, Diakonessenhuis Utrecht, Utrecht, the Netherlands; Department of Surgery, St Antonius Hospital, Utrecht, the Netherlands; Department of Medical Oncology, GROW-School for Oncology and Reproduction, Maastricht University Medical Center, Maastricht, the Netherlands; Department of Medical Psychology, Amsterdam University Medical Center location Vrije Universiteit, Amsterdam, Amsterdam, the Netherlands; Department of Public and Occupational Health, Amsterdam University Medical Center location Vrije Universiteit, Amsterdam, the Netherlands; Amsterdam Public Health Research Institute, Societal Participation and Health, Amsterdam, the Netherlands; Department of Psychosocial Research and Epidemiology, Netherlands Cancer Institute, Amsterdam, the Netherlands; Department of Psychology, University of Amsterdam, Amsterdam, the Netherlands

## Abstract

**Background:**

Cognitive problems contribute to decline in work performance. We evaluated (1) the effectiveness of basic self-management and extensive therapist-guided online cognitive rehabilitation on attainment of individually predetermined work-related goals among occupationally active cancer survivors, and (2) whether effectiveness of the programs differed for survivors with and without formal cognitive impairment.

**Methods:**

In a 3-arm randomized controlled trial (NCT03900806), 279 non–central nervous system cancer survivors with cognitive complaints were assigned to the basic program (n = 93), the extensive program (n = 93), or a waiting-list control group (n = 93). Participants completed measurements pre-randomization (T0), 12 weeks post-randomization upon program completion (T1), and 26 weeks post-randomization (T2). Mixed-effects modeling was used to compare intervention groups with the control group on goal attainment, and on self-perceived cognitive problems, work ability, and health-related quality of life.

**Results:**

Participants in the extensive program achieved their predetermined goals better than those in the control group, at short- and long-term follow-up (effect size [ES] = .49; *P* < .001; ES = .34; *P* = .014). They also had fewer recovery needs after work (ES = -.21; *P* = .011), more vitality (ES = .20; *P* = .018), and better physical role functioning (ES = .0.43 *P* = .015) than controls. At long-term follow-up, this finding persisted for physical role functioning (ES = .42; *P* = .034). The basic program elicited a small positive nonsignificant short-term (not long-term) effect on goal attainment for those with adequate adherence (ES = .28, *P* = .053). Effectiveness of the programs did not differ for patients with or without cognitive impairment.

**Conclusions:**

Internet-based therapist-guided extensive cognitive rehabilitation improves work-related goal attainment. Considering the prevalence of cognitive problems in survivors, it is desirable to implement this program.

## Introduction

With a worldwide incidence of more than 19 million new cancer cases in 2020, the burden of cancer is high (1). Cancer survivors face various survivorship issues, including cognitive problems (2-5). Cognitive problems occur in patients with brain tumors, but also in patients with non–central nervous system (CNS) disease. About 30% of non-CNS cancer survivors are confronted with cognitive problems, which are mild to moderate and predominantly include domains of learning and memory, processing speed, and executive function (6-9). Such problems may negatively impact daily life, including functioning at work (10-14).

Around 40% to 50% of new cancer patients are of working age (15,16). Cancer survivors consider being able to work an important recovery milestone and contributor to their quality of life, as it provides income and increases self-esteem (17). Within 1 year after diagnosis, more than 60% of cancer survivors manages to (partly) return to work (10). Nevertheless, a substantial number of survivors who return to work report cognitive problems that affect their work performance (18,19). To avoid loss of work performance and prevent work disability, effective treatment options for working non-CNS cancer survivors experiencing cognitive problems are warranted.

In clinical practice, neuropsychological rehabilitation is successfully used for various neurologic patient populations with cognitive impairment (20,21). It aims to improve both cognitive and noncognitive problems, and it includes elements such as psychoeducation, fatigue management, and cognitive rehabilitation. Cognitive rehabilitation can be directed at teaching the use of strategies to compensate for or cope with cognitive problems or at restoring cognitive performance itself through brain training (22). In non-CNS cancer patients, several studies have been conducted to test the efficacy of interventions based on strategy training and/or brain training, with the latter interventions generally being less effective than the former (23,24). At present, it is unclear whether interventions benefit daily life functioning as this has been evaluated rarely or only in studies with a limited sample size (25). Most studies targeted self-reported cognitive function or tested cognitive function, without a clear rationale for one over the other. Also, many studies did not differentiate between patients with self-reported cognitive complaints and tested cognitive impairment and those who only self-report problems, even though it may well be that these 2 groups require different interventions (24). Also, none of the prior studies focused on cancer survivors who experience cognitive problems at work.

In clinical rehabilitation, the success of an intervention is defined by the patient’s achievement of predetermined goals. A tool for formulating and assessing these goals is goal attainment scaling (GAS) (26). The use of GAS allows meaningful changes in the daily functioning of individual patients to be identified and measured in a standardized way (26-28).

Taken together, these insights support the execution of a multicenter randomized controlled trial (RCT): the i-WORC study (29). In this 3-arm trial, we aimed to assess the effectiveness of a basic self-management and an extensive therapist-guided online cognitive rehabilitation program on attainment of individualized work-related goals in occupationally active cancer survivors with self-perceived cognitive problems at work. We also investigated whether effectiveness of the 2 programs differs for cancer survivors with and without formal cognitive impairment, as we ultimately want to match the intensity level of a cognitive intervention to the patients’ needs.

We hypothesized that 1) cancer survivors who undergo a basic or extensive cognitive rehabilitation program will better achieve their goals compared to cancer survivors in a waiting-list control group. Furthermore, we hypothesized that 2) cancer survivors who demonstrate affected cognitive function on neuropsychological tests will specifically better achieve their goals when allocated to the extensive program compared to the basic program.

## Methods

### Research design and study sample

The i-WORC multicenter 3-arm RCT design has been published previously (29). Cancer survivors were recruited in the Netherlands from 8 hospitals and 1 occupational health service. Inclusion criteria were age between 18 and 65 years; histologically confirmed non-CNS cancer; having had systemic therapy (ie, chemotherapy, targeted agents, immunotherapy, and/or endocrine therapy) completed a minimum of 6 months before study entry (except endocrine therapy); self-reported cognitive problems at work (ie, assessed during semistructured telephone screening and specified during goal setting at baseline); occupationally active for a minimum of 8 working hours per week; fixed or temporary employment contract. Exclusion criteria were lack of basic proficiency in Dutch; serious psychiatric or neurological disorder; no Internet access; participation in comparable programs. The study was approved by the Medical Ethic Committee of the Netherlands Cancer Institute (#M18IWO) and is registered at ClinicalTrials.gov (#NCT03900806).

### Study procedures

**Figure 1.**
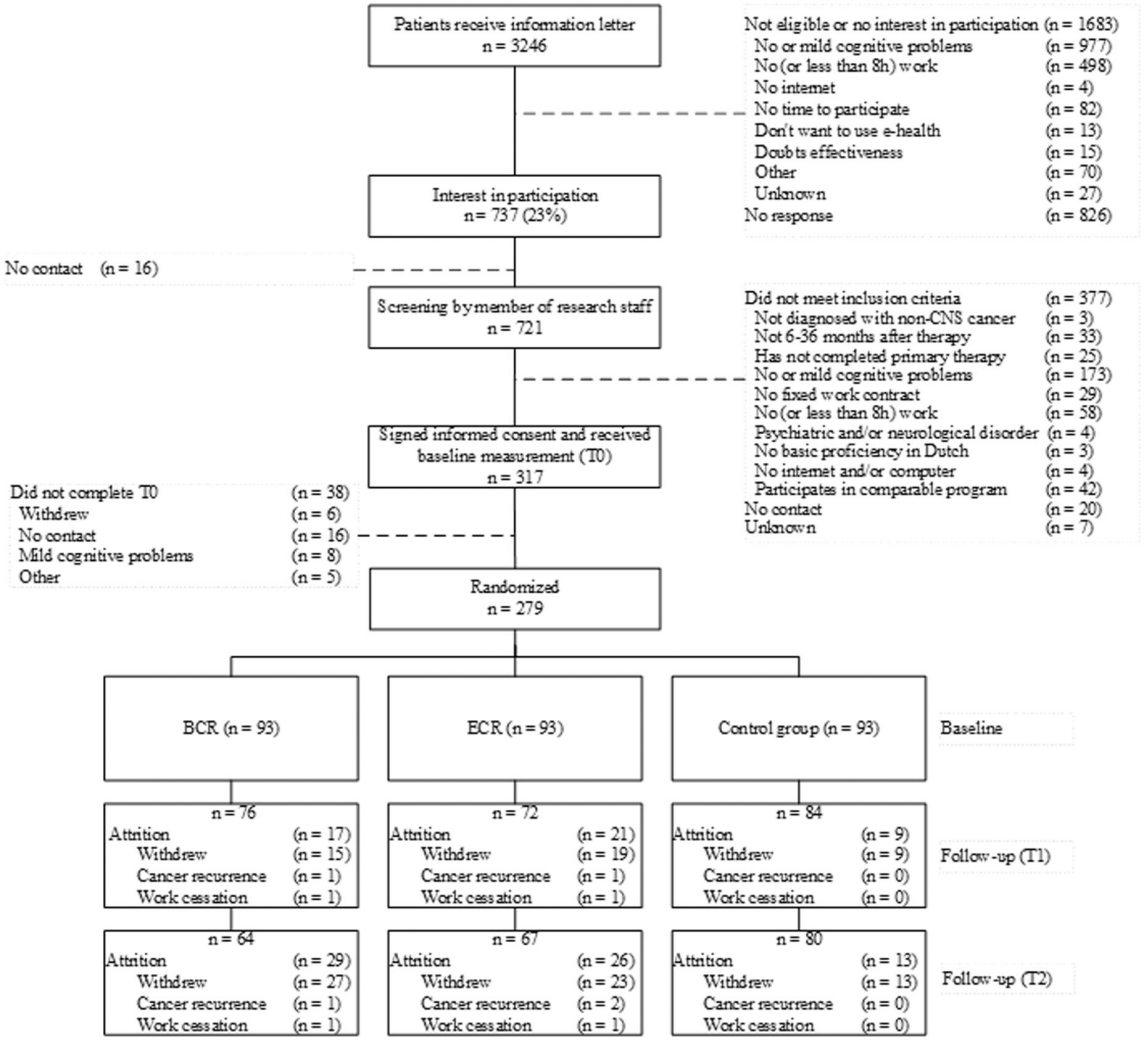
CONSORT diagram. BCR = basic cognitive rehabilitation; ECR = extensive cognitive rehabilitation.

Figure 1 provides the CONSORT diagram. Potential eligible cancer survivors were identified via hospital databases, the Netherlands Cancer Registry (NCR), and/or an occupational health service database and invited by their (occupational) physician. Cancer survivors could respond directly to the study team by e-mail, telephone, or response card. Interested survivors were screened by telephone for further eligibility by the study team. After providing informed consent and completing baseline online questionnaires and neuropsychological tests, a session (ie, initially face-to-face, later by video conference due to the COVID-19 pandemic) was scheduled with a therapist (ie, neuropsychologist or occupational therapist) to set goals. Afterwards, a research assistant used ALEA to conduct random assignment (using minimization to stratify for neuropsychological test performance). Cancer survivors were randomized to 1 of 3 groups: the basic cognitive rehabilitation (BCR) group, the extensive cognitive rehabilitation (ECR) group, or the waiting-list control group.

Participants allocated to BCR or ECR received access to a secured personal webpage, where all content of the programs was available. The 2 versions of the online program were developed based on a Dutch rehabilitation program “Niet-Rennen-Maar-Plannen” (ie, “Don’t run, but plan,” in English) in cooperation with the developers of this program. The original program is used as face-to-face cognitive rehabilitation for patient populations with cognitive impairment after acquired brain injury. The BCR and ECR were adapted for online use and modified for cancer survivors with self-perceived cognitive problems at work. Details on this development process can be found in Klaver et al. (29). Table 1 provides an overview of the content. The BCR consisted of a brief self-management program including psychoeducation, fatigue management, coping with consequences of cognitive problems, and communication strategies. The BCR did not include strategy training. The ECR included all elements of the BCR as well as strategy training modules and involved therapist guidance in which the patients’ in-session reflection and homework assignments were discussed. The strategy training modules were tailored to specific individual problems and predetermined goals (27).

**Table 1.** Outline intervention content

Modules^a^	Description	BCR	ECR
Psychoeducation	In this module, participants are acquainted with the impact of fatigue, psychological distress, and cancer treatment on cognitive functioning. The module further offers comprehensive insights into cognition and its various domains. Additionally, this module introduces the operational methodology of the rehabilitation program.	▪	▪
Fatigue management	In the first part of this module, fatigue is explained, including factors that can contribute to its persistence. Participants gain insights into their fatigue levels and how daily activities affect their fatigue through registration tasks. In the second part of the module, participants learn and practice strategies to improve their energy balance.	▪	▪
Cognitive behavioral therapy	This module explores the association between negative beliefs and cognitive problems. Participants identify and examine their negative beliefs about cognitive functioning through registration tasks. Subsequently, cognitive behavioral therapy techniques are employed to replace these negative beliefs with more functional beliefs.	▪	▪
Communication	In this module, participants are encouraged to consider whether they would like to disclose their cognitive problems at work. They are guided to formulate the message they intend to convey. Additionally, attention is devoted to the manner in which the message is conveyed.	▪	▪
Strategy training: Information processing	The first part of this module explains how information processing works, including different types of difficulties that can arise. Participants gain insight into their information processing difficulties through registration tasks. In the next part of the module, participants learn and practice strategies to effectively manage information processing difficulties in their daily lives.		▪
Strategy training: memory	The first part of this module explains how memory works, focusing on the differences between working memory and long-term memory. Participants gain understanding of these memory processes and identify their own memory difficulties through registration tasks. In the second part of the module, participants learn strategies and practice them to effectively address memory difficulties in their daily lives.		▪
Strategy training: executive function	The first part of this module provides information on executive functions, explaining their nature and importance. Through registration tasks, participants identify their own challenges in executive functioning, gaining a comprehensive understanding of their individual difficulties. In the second part of the module, participants learn strategies to effectively manage executive functioning difficulties in their daily lives.		▪
Therapist guidance	Cognitive therapists offered online weekly guidance to participants through their personal accounts on the Internet platform. This guidance was facilitated through the use of assignment response fields and/or a messaging system.		▪

a The modules contained various features to enhance the learning experience, including infographics, informative videos, animations, and assignments.

All participants completed measurements at baseline (T0) before randomization, 12 weeks after randomization upon program completion (T1), and at 26 weeks after randomization (T2). Measurements were performed online via secure websites. Rehabilitation goals were set at baseline and evaluated in a separately scheduled telephone session with all participants at T1 and T2.

### Study measures

Sociodemographic (ie, age, gender, and education) and work-related characteristics (ie, employment sector, years of work experience, working hours, and days per week according to employment contract) were obtained via questionnaires. Clinical information was obtained via questionnaires. Information on received (and future) treatment(s) was obtained via questionnaires, and, if missing, substituted with information from the NCR. Month and year of diagnosis was obtained via the NCR.

Table 2 provides an overview of primary and secondary outcome measures. GAS (26-28) was used to assess attainment of individualized work-related rehabilitation goals (primary outcome). Goals were set at baseline (T0), using the 6-point Goal Attainment Scale (GAS) on personal outcome (-3, goal achievement worse; -2, same as before; -1, partially achieved; 0, achieved; 1, exceeded, and 2, greatly exceeded). Together with the therapist, each participant formulated 2 or 3 goals and defined 6 outcome levels per goal. The evaluation of goal attainment followed a fixed process of registration and reporting of tasks, and the translation of the results into scales. The Supplementary Methods (available online) provide a description of the formulation and evaluation of goals. All team members and therapists followed a training in GAS methodology. Quality checks were performed to assure fidelity to the GAS protocol. Secondary outcomes were measured using questionnaires assessing self-reported work ability (Work Ability Index) (30,31), work functioning (Work Role Functioning Questionnaire 2.0) (32), cognitive complaints at work (Cognitive Symptom Checklist-Work Dutch Version) (33), need for recovery after work (experience and assessment of work questionnaire) (34), and health-related quality of life (Short Form-36) (35,36). Cognitive performance was assessed at baseline using an online neuropsychological test battery (Amsterdam Cognition Scan; see Supplementary Table 1, available online) (37,38). As our intervention was directed at better functioning in daily life, we did not evaluate changes in tested cognitive function over time.

**Table 2.** Outcome measures

Test Name	Items, range, subscales	Variable measured
Primary outcome		
Goal Attainment Scaling (GAS)	2 or 3 personalized treatment goals	Goal Attainment Scaling
	6-point scale (range: -3 to 2)	
	Score: 0–100 (T-score)^a^	
Secondary outcomes		
Cognitive Symptom Checklist-Work, Dutch version (CSC-W DV)	19 items	Cognitive problems
	5-point scale	
	Score: 0–100^b^	
Work Ability Index (WAI)	1 item, 10-point scale	Current work ability compared to lifetime best work ability
	Score: 1–10^a^	
Work Role Functioning Questionnaire (WRFQ)	27 items	Work functioning
	5-point scale	
	Subscales: work scheduling & output demands (WSOD), mental & social demands (MSD), flexibility demands (FD), physical demands (PD)	
	Total score: 0–100^a^	
	Subscale scores: 0–100^a^	
Experience and assessment of work questionnaire (VBBA)	11 items (subscale)	Need for recovery
	Dichotomous scale (yes/no)	
	Total score: 0–100^b^	
Short Form-36 (SF-36)	36 items	Health-Related Quality of Life
	Dichotomous and 3- to 6-point scales	
	Subscales: general health perceptions (GH), vitality (V), physical role functioning (PR), emotional role functioning (ER), mental health (MH), social role functioning (SR), physical functioning (PF), and bodily pain (BP)	
	Subscale scores: 0–100^a^	

a Higher scores indicate better performance and/or well-being.

b Higher scores indicate worse performance and/or well-being.

### Sample size calculation and statistical methods

With 65 evaluable patients per group and an alpha of 0.05, the study will have 80% power to detect an effect size of f is 0.2 for the primary effect of the intervention between the intervention groups versus the waitlist control group. To perform subgroup analysis, the sample size should be inflated fourfold (29,39). Therefore, we aim to evaluate 87 patients per group (39). Analysis of Variance or χ^2^ tests were used to compare baseline characteristics of the groups. Questionnaire scores were calculated according to published scoring algorithms (29). For each participant, an overall GAS T-score that reflects the extent to which patients’ goals were attained was computed per time point using the following algorithm (29):

$$T\;=\;50\;+\;\frac{10\sum wixi}{\sqrt{(1-\rho )\sum wi²+\rho \left(\sum wi\right)²}}$$

w_i_ is the weight assigned to goal_i_ and was assigned 1 for all goals since goals were considered equally relevant. x_i_ is the original score for goal_i_ ranging from −3 to +2. ρ is the estimated correlation between goal scores and was set at 0.3.

Scores on neuropsychological tests were converted into age-adjusted *z*-scores (mean = 0, standard deviation = 1) using normative data (37). Affected cognitive performance was determined as a *z*-score of less than or equal to -1 on at least 2 out of 10 tests from different cognitive domains (29).

To address the first hypothesis, we conducted baseline to follow-up analyses (T0-T1 and T0-T2) for the primary outcome (GAS) using mixed-effects models with an unstructured covariance structure (40). For secondary outcomes (ie, Work Ability Index [WAI], Work Role Functioning Questionnaire [WRFQ], Cognitive Symptom Checklist-Work Dutch Version [CSC-W DV], Experience and assessment of work questionnaire [VVBA], and Short Form-36 [SF-36]), we conducted baseline to follow-up analyses (T0-T1 and T0-T2) using mixed-effects models with a random intercept and an autoregressive covariance structure. Within each mixed-effect model, the control group was the reference category. Group, time, and the interaction of group by time were entered as independent variables. We adjusted for non-ignorable dropout (41) as rates of missing data were significantly different between groups, ie, more participants were missing in the intervention groups compared to the control group (attrition rates at T1: BCR n = 17; ECR n = 21; control group n = 9 and at T2 BCR n = 29; ECR n = 26; control group n = 13). This allowed evaluation of the contribution of missing data patterns to the outcome by adding the missing data pattern and its interaction with both group and time to the model. To address the second hypothesis, we added neuropsychological test performance (ie, affected yes/no) and the interaction of group by time by neuropsychological test performance as independent variables into the model.

The *P* value for statistical significance (2-sided) of overall model effects was set at .05. Differences in change from baseline to follow-up between groups were accompanied by effect sizes (ES). ES was calculated based on the between-group difference in mean change and pooled SD of the intervention and control groups. An ES of 0.2 was considered small, 0.5 moderate and clinically relevant (42), and 0.8 large. All analyses were done on intention-to-treat (ITT) basis. Per protocol (PP) analyses were performed for the primary outcome in participants who started the program and met criteria for minimal adherence. Since there are no clear guidelines for determining minimum intervention adherence, we opted for a relatively low requirement of completion of at least 70% of 2 modules.

## Results

### Sample characteristics

Recruitment took place between November 2019 and September 2021. Invitations were sent to 3246 patients who met the medical eligibility criteria. The CONSORT diagram (Figure 1) provides an overview of the recruitment process. In total, 279 cancer survivors completed the baseline measurement and were randomly assigned to the BCR group (n = 93), ECR group (n = 93), or the waitlist control group (n = 93). Completion rates of the primary outcome were 83% at T1 (n = 232) and 76% at T2 (n = 211).

Sociodemographic, treatment-related, and work-related characteristics are presented in Table 3.

**Table 3.** Baseline sociodemographic and clinical characteristics of cancer survivors

	All participants	BCR group	ECR group	Control group
	n = 279	n = 93	n = 93	n = 93
Sociodemographic characteristics				
Age (years): mean (SD)/range	49.1 (8.4)/27-65	48.6 (9.1)/29-65	49.2 (8.6)/27-63	49.4 (7.4)/30-63
Gender, n (%)				
Female	233 (83)	76 (82)	79 (85)	78 (84)
Male	46 (17)	17 (18)	14 (15)	15 (16)
Marital status, n (%)				
Single	42 (15)	12 (13)	13 (14)	17 (18)
Married	151 (54)	54 (58)	50 (54)	47 (51)
Living with partner	62 (22)	19 (20)	21 (23)	22 (24)
Divorced	20 (7)	6 (7)	7 (8)	7 (8)
Widowed	4 (1)	2 (2)	2 (2)	0 (0)
Education, n (%)				
None/primary/lower vocational	5 (2)	2 (2)	2 (2)	1 (1)
Secondary school/vocational education	88 (32)	27 (29)	27 (29)	34 (37)
Upper secondary school/upper vocational education/university	186 (67)	64 (69)	64 (69)	58 (62)
Clinical and treatment-related characteristics at baseline				
Cancer type, n (%)				
Breast	195 (70)	63 (68)	67 (72)	65 (70)
Digestive—colon	9 (3)	3 (3)	4 (4)	2 (2)
Digestive—other	6 (2)	1 (1)	2 (2)	3 (3)
Head and neck	5 (2)	4 (4)	1 (1)	0 (0)
Hodgkin lymphoma	6 (2)	2 (2)	2 (2)	2 (2)
Non-Hodgkin lymphoma	8 (3)	2 (2)	3 (3)	3 (3)
Leukemia	6 (2)	4 (4)	1 (1)	1 (1)
Respiratory	6 (2)	3 (3)	0 (0)	3 (3)
Ovarian	3 (1)	1 (1)	1 (1)	1 (1)
Prostate	6 (2)	2 (2)	4 (4)	0 (0)
Testis	7 (3)	2 (2)	0 (0)	5 (5)
Cervix	8 (3)	5 (5)	3 (3)	0 (0)
Dermatologic	4 (1)	0 (0)	1 (1)	3 (3)
Other	10 (4)	1 (1)	4 (4)	5 (5)
Metastasis, n (%)				
Metastatic disease	16 (6)	7 (8)	5 (5)	4 (4)
No metastasis	263 (94)	86 (92)	88 (95)	89 (96)
Time since diagnosis (years): mean (SD)	2.6 (1.2)	2.8 (1.5)	2.5 (0.9)	2.6 (1.1)
Treatment, n (%)				
Surgery	240 (86)	75 (81)	81 (87)	84 (90)
Chemotherapy	244 (88)	78 (84)	82 (88)	84 (90)
Immunotherapy/targeted therapy	57 (20)	14 (15)	22 (24)	21 (23)
Hormonal therapy	128 (46)	48 (52)	48 (52)	32 (34)
Radiotherapy	195 (70)	65 (70)	67 (72)	63 (68)
Work-related characteristics				
Sector, n (%)*				
Business and financial	30 (11)	18 (19)	7 (8)	5 (5)
Education	34 (12)	11 (12)	12 (13)	11 (12)
Industry	11 (4)	4 (4)	5 (5)	2 (2)
Health care	88 (32)	22 (24)	34 (37)	32 (34)
Trade	13 (5)	1 (1)	4 (4)	8 (9)
Public services	29 (10)	13 (14)	5 (5)	11 (12)
Culture, recreation	18 (7)	5 (5)	7 (8)	6 (7)
Other	56 (20)	19 (20)	19 (20)	18 (19)
Working hours per week: mean (SD)/range^a^	29.6 (8.1)/8-40	30.8 (8.1)/12-40	29.2 (8.4)/8-40	28.8 (7.9)/8-40
Employment type				
Fixed	245 (89)	79 (85)	83 (89)	83 (89)
Temporary	21 (8)	6 (7)	6 (7)	9 (10)
Other	13 (5)	8 (9)	4 (4)	1 (1)
Shift work, n (%)				
Yes	58 (21)	21 (23)	13 (14)	24 (26)
No	221 (79)	72 (77)	80 (86)	69 (74)

a To promote patient inclusion, the eligibility threshold for working hours was revised from requiring a minimum of 12 hours per week to 8 hours per week. There were no statistically significant differences between groups at baseline on any other variable. BCR = basic cognitive rehabilitation; ECR = extensive cognitive rehabilitation; n = number; SD = standard deviation.

* *P* < .05.

A total of 279 cancer survivors (46 men and 233 women) with a mean age of 49.1 years (SD 8.4; range 27-65 years) were included. Breast cancer was the most prevalent cancer type among female survivors (n = 195; 84%). Testis carcinoma was the most prevalent cancer type among male survivors (n = 7; 15%). Mean time since diagnosis at baseline was 2.6 years (SD = 1.2 years; range 1.0-11.1 years). Cancer survivors worked on average 29.6 hours/week (SD = 8.1 hours; range 8-40 hours).

### Primary outcome: Goal attainment

**Figure 2.**
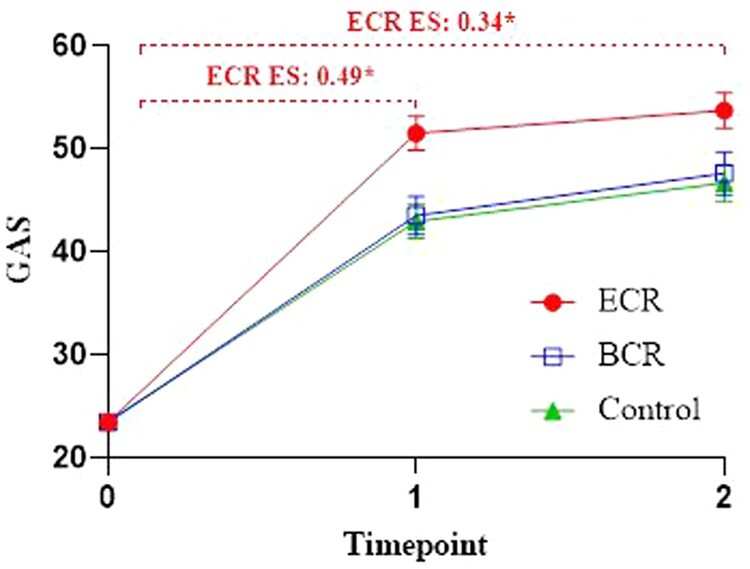
Mean values (including standard errors of measurement) and effect sizes of goal attainment scores over time. ES = effect size; GAS = Goal Attainment Scaling; ECR = extensive cognitive rehabilitation; BCR = basic cognitive rehabilitation. *P < .05. Note: due to a lack of variation in GAS T0 scores (SD = 1), ES for GAS were calculated based on the *t* test statistic: (2*t)/(√df); small, 0.2; moderate, 0.5; large, 0.8.

Statistically significant improvements in goal attainment over time were observed for all 3 groups (Figure 2). Specific contrasts showed that participants in the ECR group achieved their predefined goals significantly better than participants in the control group at both short-term (T0-T1) and long-term (T0-T2) follow-up (ES=.49; *P* < .001; ES=.34; *P* = .014, respectively). Participants in the BCR group did not report significantly better goal attainment compared to the control group at both short-term (T0-T1) and long-term (T0-T2) follow-up (ES=.06; *P* = .66; ES=.01; *P* = .92, respectively).

#### PP analyses

Intervention adherence rates were 90% (n = 71) in the ECR group and 70% (n = 59) in the BCR group. Results from the PP analyses showed that participants in the ECR group achieved their predefined goals significantly better at short and long-term than the control group (ES= .48; *P* = .001 and ES= .32; *P* = .029, respectively). For the BCR group, we found nonsignificant better goal attainment compared to the control group at short term (ES=.28; *P* = .053), and no statistically significant differences at long term (ES=.23; *P* = .12).

### Secondary outcomes

Results of the ITT analyses are presented in Tables 4 and 5. At short-term follow-up, the ECR group had statistically significantly less recovery need after work (VBBA: ES = -.21; *P* = .011), more vitality (SF_V: ES = .20; *P* = .018), and better physical role functioning (SF_PR: ES = .43 *P* = .015) than the control group. At long-term follow-up, this finding only persisted for physical role functioning (ES = .42; *P* = .034). Both at short-term and long-term follow-up, no statistically significant group differences over time were observed between the control group and intervention groups for self-reported cognitive complaints at work (CSC-W-DV), work ability (WAI), work functioning (WRFQ), cognitive symptoms (CSC), general health (SF_GH), emotional role functioning (SF_ER), mental health (SF_MH), social role functioning (SF_SR), physical functioning (SF_PF), and bodily pain (SF_BP).

**Table 4.** Mean values at baseline, short-term, and long-term follow-up and between-group differences for the mixed-effect models of work-related outcome measures

	Baseline (T0)		Short-term follow-up (T1)		Long-term follow-up (T2)		Between-group difference T0-T1				Between-group difference T0-T2			
Secondary outcomes	N	Mean SD	N	Mean SD	N	Mean SD	Estimate	95% CI	*P*	Effect size	Estimate	95% CI	*P*	Effect size
CSC-Work (*P*** **= .83)^a^														
ECR	91	43.58	63	35.86	62	34.31	−0.39	−3.33 to 2.55	.80	−0.03	−1.08	−4.42 to 2.26	.53	−0.24
		13.05		12.14		12.59								
BCR	90	42.83	62	36.31	60	35.84	−1.49	−4.44 to 1.47	.32	−0.11	−1.65	−5.01 to 1.71	.33	−0.08
		12.84		13.66		13.16								
Control^b^	89	43.97	69	38.86	75	38.02								
		12.10		12.28		13.31								
WAI (*P*** **= .69)^a^														
ECR	93	6.13	71	6.80	63	6.79	0.13	−0.26 to 0.52	.52	0.06	−0.046	−0.50 to 0.41	.84	0.12
		1.42		1.42		1.72								
BCR	93	6.16	70	6.67	61	6.49	0.17	−0.22 to 0.56	.38	0.08	−0.12	−0.58 to 0.34	.61	−0.03
		1.42		1.53		1.99								
Control^b^	93	6.23	85	6.64	77	6.82								
		1.29		1.38		1.60								
WRFQ (*P*** **= .56)^a^														
ECR	91	69.79	70	78.88	58	78.58	2.28	−0.94 to 5.50	.17	0.11	0.98	−3.12 to 5.07	.64	0.22
		14.35		12.61		14.09								
BCR	91	70.51	69	76.56	54	77.16	−0.35	−3.57 to 2.87	.83	−0.02	−0.49	−4.63 to 3.66	.82	0.11
		14.91		16.70		15.75								
Control^b^	93	69.01	81	74.13	68	76.28								
		13.75		14.69		14.90								
VBBA (*P*** **= .049*)^a^														
ECR	93	72.92	71	58.90	63	61.76	−8.41	−14.90 to 1.92	.011*	−0.21	−0.52	−8.25 to 7.21	.89	−0.10
		22.67		28.62		30.60								
BCR	93	68.52	70	58.70	61	55.74	−3.42	−9.92 to 3.08	.30	−0.09	−2.72	−10.51 to 5.06	.49	−0.16
		25.94		30.26		31.16								
Control^b^	93	68.43	85	64.28	77	59.98								
		24.88		25.59		29.36								

Reported are the model-based means and standard deviations. Models were adjusted for non-ignorable dropout. BCR = basic cognitive rehabilitation; ECR = extensive cognitive rehabilitation; CI = Confidence Interval; CSC-Work = Cognitive Symptom Checklist—Work; WAI = Work Ability Index; WRFQ = Work Role Functioning Questionnaire; VBBA = experience with work; SD = standard deviation. T0 = baseline; T1 = mid-treatment; T2 = post-treatment.

a *P* value of the overall interaction effect between group and time.

b Control group is reference group.

* *P* < .05,

** *P* < .001.

**Table 5.** Mean values at baseline, short-term, and long-term follow-up and between-group differences for the mixed-effect models of health-related quality of life (SF-36 subscales)

	Baseline (T0)		Short-term follow-up (T1)		Long-term follow-up (T2)		Between-group difference T0-T1				Between-group difference T0-T2			
SF-36 subscales	N	Mean SD	N	Mean SD	N	Mean SD	Estimate	95% CI	*P*	Effect size	Estimate	95% CI	*P*	Effect size
SF GH (*P* = .45)^a^														
ECR	93	57.04	70	60.57	63	58.17	3.73	−0.50 to 7.96	.084	0.10	1.22	−3.78 to 6.22	.63	0.00
		18.08		19.57		19.76								
BCR	93	59.35	70	60.79	61	59.67	0.66	−3.58 to 4.90	.76	0.01	0.39	−4.64 to 5.43	.88	0.00
		17.01		17.77		17.75								
Control^b^	93	58.44	85	59.94	77	60.84								
		16.13		17.55		17.72								
SF V (*P* = .037*)^a^														
ECR	93	48.33	70	56.07	63	56.27	4.95	0.87 to 9.02	.018*	0.20	2.47	−1.92 to 6.86	.27	0.09
		15.28		15.44		16.06								
BCR	93	48.92	70	54.21	61	51.23	1.95	−2.13 to 6.03	.35	0.13	−2.78	−7.20 to 1.64	.22	−0.07
		17.26		19.29		19.31								
Control^b^	93	51.56	85	54.06	77	56.30								
		13.99		16.88		16.81								
SF PR (*P* = .055)^a^														
ECR	93	38.17	70	65.71	63	66.27	15.90	3.11 to 28.70	.015*	0.43	15.11	1.16 to 29.06	.034*	0.42
		36.41		41.52		42.40								
BCR	93	45.16	70	56.07	61	63.93	2.61	−10.20 to 15.43	.69	0.11	11.45	−2.60 to 25.50	.11	0.26
		41.91		44.32		40.45								
Control^b^	93	46.51	85	54.71	77	56.91								
		40.48		40.55		40.07								
SF ER (*P* = .58)^a^														
ECR	93	58.78	70	68.57	63	71.42	−2.97	−16.12 to 10.18	.66	0.07	−11.55	−26.26 to 3.16	.12	−0.08
		43.52		38.02		38.27								
BCR	93	57.71	70	70.48	61	75.41	0.59	−12.59 to 13.77	.93	0.09	−6.78	−21.60 to 8.04	.37	−0.04
		40.87		39.54		35.95								
Control^b^	93	58.06	85	67.45	77	77.92								
		42.82		40.16		34.88								
SF MH (*P* = .44)^a^														
ECR	93	67.53	70	71.26	63	71.75	2.65	−1.51 to 6.80	.21	0.24	0.81	−3.78 to 5.40	.73	0.16
		15.41		15.02		17.04								
BCR	93	67.18	70	72.57	61	71.61	3.86	−0.30 to 8.02	.069	0.32	1.26	−3.36 to 5.89	.59	0.21
		17.78		15.46		17.22								
Control^b^	93	70.41	85	70.54	77	72.31								
		13.88		16.42		16.02								
SF SR (*P* = .62)^a^														
ECR	93	64.38	70	71.43	63	75.00	1.68	−4.89 to 8.26	.61	0.17	4.31	−2.70 to 11.32	.23	0.26
		18.79		22.13		21.18								
BCR	93	68.01	70	72.14	61	74.59	−2.29	−8.88 to 4.29	.49	0.06	−0.64	−7.70 to 6.42	.86	0.11
		20.81		21.82		22.00								
Control^b^	93	66.94	85	71.76	77	72.24								
		19.73		19.97		21.42								
SF PF (*P* = .52)^a^														
ECR	93	81.51	70	83.14	63	86.03	−0.40	−4.10 to 3.31	.83	−0.11	2.66	−0.76 to 6.09	.13	0.04
		13.73		16.84		13.89								
BCR	93	84.78	70	85.00	61	85.57	−0.38	−4.10 to 3.33	.84	−0.17	1.07	−2.38 to 4.53	.54	−0.10
		14.01		14.04		13.91								
Control^b^	93	81.34	85	83.53	77	83.77								
		12.56		14.49		12.70								
SF BP (*P* = .99)^a^														
ECR	93	85.09	70	86.29	63	85.75	−0.53	−5.53 to 4.47	.84	−0.17	−0.26	−5.35 to 4.83	.92	−0.15
		16.37		15.67		18.04								
BCR	93	85.71	70	86.79	61	83.98	0.19	−4.82 to 5.20	.94	−0.19	−0.98	−6.12 to 4.15	.71	−0.24
		15.87		15.85		16.87								
Control^b^	93	84.61	85	86.21	77	84.77								
		15.44		14.49		16.44								

Reported are the model-based means and standard deviations. Models were adjusted for non-ignorable dropout. BCR = basic cognitive rehabilitation; ECR = extensive cognitive rehabilitation; CI = confidence interval; SF GH = Short Form—General Health Perceptions; SF V = Short Form—Vitality; SF PR = Short Form—Physical Role Functioning; SF ER = Short Form—Emotional Role Functioning; SF MH = Short Form—Mental Health; SF SR = Short Form—Social Role Functioning; SF PF = Short Form—Physical Functioning; SF BP = Short Form—Bodily Pain; SD = standard deviation. T0 = baseline; T1 = mid-treatment; T2 = post-treatment.

a *P* value of the overall interaction effect between group and time.

b Control group is reference group.

* *P* < .05,

** *P* < .001.

### Cognitive performance pre-intervention

**Figure 3.**
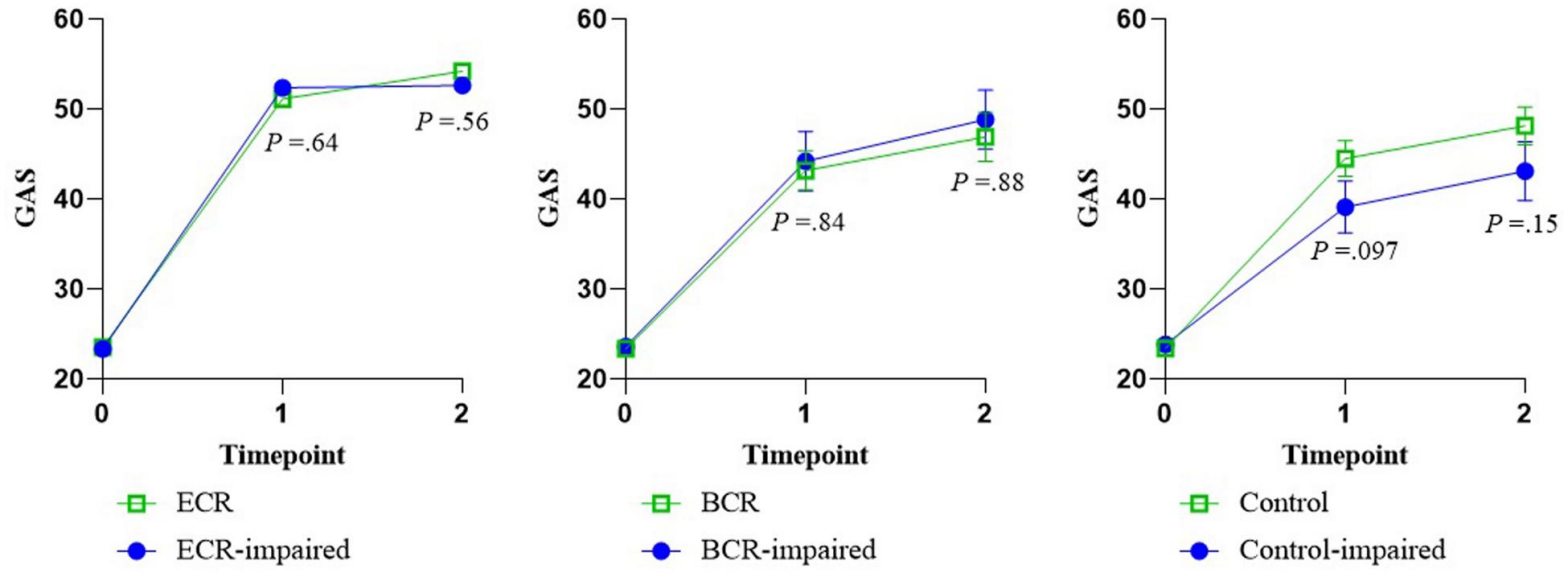
Mean values (including standard errors of measurement) of goal attainment scores over time grouped on cognitive performance preintervention. GAS = Goal Attainment Scaling; ECR = extensive cognitive rehabilitation; BCR = basic cognitive rehabilitation. Note: The figure presents the *P* values of the interaction between time and neuropsychological test performance per study group.

No statistically significant group by time by neuropsychological test performance interaction was observed for GAS between cancer survivors in the BCR and ECR at T1 (β = -.83; 95% CI = -10.97 to 9.31; *P* = .87) and T2 (β = 3.20; 95% CI = -7.74 to 14.15; *P* = .56) (Figure 3). This indicates that effects of ECR compared to BCR over time did not differ significantly between cancer survivors with or without affected cognition at baseline.

## Discussion

As cancer- and cancer-treatment-related cognitive problems impact cancer survivors’ daily functioning, including work functioning, interventions to improve daily life functioning are crucial. The present study indicates that extensive therapist-guided online cognitive rehabilitation provides an effective and clinically relevant treatment for achievement of individually predetermined work-related goals in cancer survivors who experienced cognitive problems at work. It also has positive effects on the need for recovery after work, vitality, and physical role functioning, possibly attributed to improved management of fatigue in daily life. The effect on goal attainment was maintained, albeit smaller, after longer follow-up. The basic self-management program elicited a small beneficial short-term (not long-term) effect on goal attainment compared to the control group, but only for those compliant to the self-management program, underscoring the importance of following the program carefully (24,43-45). Given that self-help interventions are more affordable and scalable compared to therapist-guided interventions (46), it is important to explore characteristics of minimally adherent survivors who may benefit from the self-management intervention and to design strategies to improve adherence. Furthermore, both cancer survivors with and without affected cognitive performance achieved their goals better when allocated to the extensive program compared to the basic program, while we expected an added benefit of the extensive program for those formally classified as cognitively affected. This suggests that the extensive program is an effective treatment approach for both groups of cancer survivors.

Interestingly, despite differences in success rate between study groups, improvement over time in goal attainment was observed for all groups, including the control group. Potentially, a neuropsychological assessment with feedback and identification of work-related situations for which improvement of functioning is most important has a therapeutic effect (47,48). Perhaps awareness of key problems at work can in itself lead to change in behavior. Conversely, this awareness may have triggered a response shift that potentially explains the lack of improvement in self-perceived cognitive complaints observed in this study. It is noteworthy that controls with impaired cognitive performance seem to achieve their goals less well (although not statistically significant) than controls without impaired cognitive performance, potentially suggesting higher intervention needs for those with impaired functioning.

Strengths of the present study include its longitudinal randomized controlled design, adequate power, Internet-based delivery of interventions, and a primary outcome that reflects functioning in daily life. This trial also has limitations. First, the basic and extensive program differed in several features (ie, therapist guidance and strategy training). It was therefore not possible to specify which feature is associated with effectiveness. Second, the study population consisted mostly of highly educated breast cancer survivors, with 32% working in health care. As such, the study sample does not represent the population of working cancer survivors. Third, there was differential loss to follow-up. Fewer participants were available for follow-up measurement in the intervention groups compared to the control group. The primary reason for attrition in the intervention groups was time constraints (discussed further in a process evaluation, to be published elsewhere). Seemingly, attendance in a rehabilitation program combined with assessment measurements was burdensome for a subgroup of participants. However, all group comparisons were corrected for differential missing data patterns, and these corrections did not affect conclusions.

This study shows that an online therapist-guided extensive cognitive rehabilitation program is effective for cancer survivors with cognitive problems at work. An online self-management basic cognitive rehabilitation program may represent a reasonable alternative, but only when adherence is sufficient. Given the high prevalence of cognitive problems in occupationally active cancer survivors, efforts should be undertaken to make the extensive program easily accessible for cancer survivors.

## Supplementary Material

pkad110_Supplementary_Data

## Data Availability

Upon reasonable request, the dataset generated during this study is available from the corresponding author.
