# Supplementary material for: Internet-based cognitive rehabilitation for working cancer survivors: results of a multicenter randomized controlled trial
Source: JNCI Cancer Spectr. 2024 Jan 25;8(1):pkad110. doi: 10.1093/jncics/pkad110 (PMC10868395; doi:10.1093/jncics/pkad110)
Supplement: pkad110_Supplementary_Data [file pkad110_supplementary_data.pdf]

Supplementary Methods

Goal Attainment: formulation and evaluation of goals

The study team including the therapists underwent an extensive training in GAS methodology.

Formulation of goals at baseline: participants formulated two to three work-related goals in collaboration with their therapist at baseline (T0). For each goal attainment scale (GAS), 6 outcome levels were defined (-3, achievement of the goal after training is worse; -2, achievement of the goal is the same; 1, partial achievement of the goal; 0, achievement of the goal; 1, exceeding the goal, and 2, greatly exceeding the goal).

Goals were formulated according to the following quality standards:

- The formulated goals were relevant to the participant.
- The goals were specific, measurable, achievable, realistic and time-bound (SMART).
- Each goal contained a maximum of one variable.
- The goals were formulated at the level of daily functioning (i.e., participation or activities in daily life).

Evaluation of goals at follow-up: the evaluation of goal attainment was conducted in a standardized manner. Leading up to the T1 and T2 assessments, participants received instruction by phone by a member of the study team to start the registration tasks (see Table Supplementary Methods). The individual registration tasks were sent to the participants per email. Once the registration was completed, participants received a phone call from one of the therapists. The registration results provided by the participants were then translated by the therapist into the goal attainment scales. Participants were not involved in this last step of the process. The process was monitored for quality control by members of the study team.

Table Supplementary Methods: Examples of Goal Attainment Scales

Example 1.		
**Setting**		When executing a work action, I forget what I was going to do. For example, when I'm sending an e-mail, I forget what content I wanted to write by the time I opened the mail.
**Registration task**		During a workweek (i.e., 3 working days of 8 hours) I score how many times I lose track of what I'm doing while performing my work.
**Outcome level**		
	**-3**	More than 10 times a week
	**-2**	10 times a week
	**-1**	7 to 9 times a week
	**0**	5 or 6 times a week
	**1**	3 or 4 times a week
	**2**	Less than 3 times a week
Example 2.		
**Setting**		In my job, I fill in application forms with my clients. Due to a lack of concentration, I have to ask my colleagues to fill in these forms.
**Registration task**		Over the course of one working week (i.e., 4 working days of 8 hours each), I record the number of pages of the application form that I complete per client. The outcome level reflects the average number of pages completed by me per client.
**Outcome level**		
	**-3**	Less than 1 page
	**-2**	1 or 2 pages
	**-1**	3 or 4 pages
	**0**	5 or 6 pages
	**1**	7 or 8 pages
	**2**	More than 8 pages
Example 3.		
**Setting**		After a day at work, I have no energy left, I'm completely exhausted. I would like to have a better work-life balance.
**Registration task**		I keep a record of the activities I do after each day's work over the course of one working week (i.e., 3 work days of 4 hours each). The following activities are awarded 1 point: cooking, vacuum cleaning, take a 20 minute walk. The following activities are awarded 2 points: reading to my children, playing with my child, talking to my husband or a friend, watching television. The outcome level reflects the average number of points scored during a working day.
**Outcome level**		
	**-3**	Less than 1 point
	**-2**	1 point
	**-1**	2 or 3 points
	**0**	4 or 5 points
	**1**	6 or 7 points
	**2**	More than 7 points

Supplementary Table 1

Supplementary Table 1. Tests of the Amsterdam Cognition Scan and their equivalent traditional tests

Online tests	Domain	Main outcome measure	Traditional equivalent
Connect the Dots I	Attention	Completion time	Trail Making Test A
Connect the Dots II	Executive function	Completion time	Trail Making Test B
Wordlist Learning	Verbal learning	Number of correctly recalled words	Rey Auditory Verbal Learning test
Reaction Speed	Processing speed	Mean reaction time	Visual Reaction Time (subtest FePsy)
Place the Beads	Planning	Number of extra moves	Tower of London, Drexel University
Box Tapping	Visuospatial memory	Number of correctly repeated sequences	Corsi Block-tapping Test
Fill the Grid	Fine motor skills	Completion time	Grooved Pegboard
Wordlist Delayed Recall	Retention of information	Number of correctly recalled words	Rey Auditory Verbal Learning test
Digit Sequences I	Attention	Number of correctly repeated sequences	WAIS Digit Span
Digit Sequences II	Working memory	Number of correctly repeated sequences	WAIS Digit Span
